# Factors influencing medication adherence among patients with severe mental disorders from the perspective of mental health professionals

**DOI:** 10.1186/s12888-021-03681-6

**Published:** 2022-01-07

**Authors:** Mengjie Deng, Shuyi Zhai, Xuan Ouyang, Zhening Liu, Brendan Ross

**Affiliations:** 1 National Clinical Research Center for Mental Disorders, and Department of Psychiatry, The Second Xiangya Hospital of Central South University, Changsha, Hunan China; 2 Faculty of Medicine, McGill University, Montreal, QC Canada

**Keywords:** Medication adherence, Severe mental disorders, Qualitative research

## Abstract

**Background:**

Medication adherence is a common issue influenced by various factors among patients with severe mental disorders worldwide. However, most literature to date has been primarily quantitative and has focused on medication adherence issue from the perspective of patients or their caregivers. Moreover, research focused on medication adherence issue in China is scarce. Present study aims to explore the influential factors of medication adherence among patients with severe mental disorders form the perspective of mental health professionals in Hunan Province, China.

**Methods:**

A qualitative study was performed in Hunan Province, China with 31 mental health professionals recruited from October to November 2017. And semi-structured interviews or focus group interviews were conducted along with audio recordings of all interviews. Interview transcripts were then coded and analyzed in Nvivo software with standard qualitative approaches.

**Results:**

Three major themes influencing medication adherence among patients with severe mental disorders were identified as: (1) attitudes towards mental disorder/treatment; (2) inadequate aftercare; (3) resource shortages.

**Conclusions:**

This qualitative study identified the factors influencing medication adherence among patients with severe mental disorders in China. As a locally driven research study, it provides practical advice on medication adherence promotion for mental health workers and suggests culturally tailored models that improve the management of patients with severe mental disorders in order to reduce economic burden on individual and societal level.

## Background

Medication adherence is defined as a patient’s medication taking behavior that complies with recommendations from healthcare providers [1]. To maintain treatment effectiveness and prevent relapse, it is important for patients with severe mental disorders, such as schizophrenia, bipolar disorder and major depressive disorder, to keep taking their medications continuously over the long term [2–4]. However, the rates of medication nonadherence among schizophrenia patients, bipolar disorder patients and patients with major depressive disorder in China were approximately 56, 48 and 51% at the beginning of 21st century, respectively [5–8]. As a result of this poor adherence, 60% of patients with severe mental disorders have experienced high rates of disability, rehospitalization and suicide [9, 10], which has increased the burden on the healthcare system [11]. Therefore, it is worthwhile to carefully examine the issue of medication adherence, which plays a key role in the management and improvement of clinical outcome in patients with severe mental disorders.

Medication adherence among patients with severe mental disorders is a complex issue determined by a multitude of factors, such as treatment effect, patient insight, attitudes toward medication, financial and emotional support from family members, income situation, side effects, cultural context, level of therapeutic alliance with therapists, and aftercare environment after discharge [12–18]. With respect to China, a shortage in the mental health workforce has also been observed by Xiang et al. [19] , which may contribute to medication nonadherence [14]. Moreover, according to the World Health Organization (WHO)’s report in 2018 [20], a lack of mental health resources, including mental health facilities, government expenditure on mental health, and the mental health workforce, is a prominent issue worldwide that negatively impacts rates of medication adherence. Although researchers have used various methodologies to study medication adherence in patients with severe mental disorders, most studies to date have focused on the perspectives of patients or their caregivers (usually their family members) [21–24]. The limitation of these papers lies in the potential omission of the opinion from other important parties in the process of care, who may also provide insights into our understanding of the issue. Mental health professionals such as psychiatrists, psychiatric nurses, psychologists, and social workers are all valuable sources to seek opinions from, as they are experienced in handling adherence issues and are also knowledgeable in identifying other contributing factors on the pharmaceutical, psychosocial, and systemic level. However, to the best of our knowledge, only two qualitative studies had focused on mental health professionals’ attitudes towards medication adherence, with one conducted in rural China [25] and the other conducted in 4 European countries (England, Germany, Italy and the Netherlands) [26]. Thus, qualitative research that explore their perspectives on medicine adherence is key to tackling this issue comprehensively.

China, one of the world’s most rapidly developing countries, also faces a serious mental health burden, of which severe mental disorders [27] constitute a significant part. In China, lifetime prevalence of schizophrenia, bipolar disorder and major depressive disorder have reached 0.7, 0.6 and 3.4%, respectively [28]. A meta-analysis, however, reported that the treatment rate of schizophrenia in psychiatric institutions was only 31% [29]. It implies that nearly 70% of schizophrenia patients did not receive treatment. The disability-adjusted life-year (DALYs) rate per 100,000 of schizophrenia and bipolar disorder are 322.8 and 109.3 respectively [27]. Considering factors such as disparities in mental health services, mental health workforce scarcity, and weaknesses in community-based management, medication adherence is a key factor that determines performance in managing severe mental disorders as well as an important indicator of the overall quality of care. To the best of our knowledge, qualitative research focused on the medication adherence situation in China is insufficient. Using a qualitative approach with semi-structured interviews and focus groups, this study explores influential factors of medication adherence in Hunan, China, with a particular focus on the perspective of mental health professionals from urban and rural areas. The study may also provide useful information for improving the management of severe mental disorders.

## Methods

### Participants

Participants were enrolled by mental health professionals in mental health institutions from October to November 2017 and included psychiatrists based in mental health hospitals, directors of psychosocial rehabilitation services (also called clubhouses), researchers of public mental health, social workers, and psychological counselors. According to previous studies [26, 30], we defined that all participants should have at least 2 years working experience related to mental health. We conducted in-depth semi-structured interviews and focus groups, and performed participant observation as well. After the first interview, we identified the next participant(s) by means of the snowball sampling method [31]. The number of participants enrolled was based on data saturation, that is, data collection was terminated once no new themes were identified and a theoretical end point of saturation was reached [32, 33]. Ultimately, a total of 31 participants were recruited: 14 psychiatrists, 2 researchers of public mental health, 7 directors of clubhouses, 7 psychological counselors, and 1 social worker were included. In addition, we conducted 22 individual interviews as well as 2 focus groups (including 3 psychiatrists and 6 psychological counselors). Detailed demographic information of participants is displayed in Table 1. All participants were subsequently numbered during the process of data analysis to aid in organization and analysis.

**Table 1:** Respondent information

Demographic characteristics	Number of participants	Percentage (%)
**Sex**		
Female	18	58.06
Male	13	41.94
**Work**		
Psychiatrist	14	45.16
Director of clubhouse	7	22.58
Psychological counselor	7	22.58
Researcher of public mental health	2	6.45
Social worker	1	3.23
**Workplace**		
Hospital	14	45.16
Rehabilitation service	8	25.81
University	7	22.58
Public health institution	2	6.45
**Location**		
Urban area	23	74.19
Rural area (western part of Hunan Province)	8	25.81

### Data collection

One researcher conducted all interviews in Mandarin and each interview averaged 45-60 minutes. Observational field notes were collected as well. In addition, all interviews were audio-recorded and were conducted with a list of pre-determined open-ended questions (Table 2). The audio records of all interviews were transcribed verbatim in Mandarin by other researchers unfamiliar with this study. All of the participants signed informed consent before the interview. The study was approved by the ethics committee of the Second Xiangya Hospital of Central South University.

**Table 2:** Interview guide

**Open-ended questions about medication adherence among patients with severe mental disorders**	
**1.** What is your current position at work and what are your clinical responsibilities?	
**2.** In your daily work, have you ever encountered problems that patients with severe mental disorders, such as schizophrenia, bipolar disorder and major depressive disorder, discontinue the medications prescribed by psychiatrists?	
**3.** Do you suggest/help these patients to continue their medications? How do you do that?	
**4.** Do you face with any difficulties when suggesting/helping these patients to continue medications?	
**5.** Are there any other people (family members/friends/other caregivers/other healthcare providers?) assisting these patients taking medications? How do they help?	
**6.** Besides mental health hospitals or psychiatry department in general hospitals, are there any other mental health services that can help these patients continue medication treatment? What roles do they play specifically?	
**7.** How is community-based mental health care organized to help these patients continue medication?	
**8.** Did the government propose any policy or implement any programs to help these patients continue medication treatment? How do they help?	
**9.** Are there any religious/cultural customs that may influence these patients to continue medication treatment?	
**10.** Besides the points you just mentioned, what other situations/factors can you think of that may influence these patients to continue medication treatment?	
**11.** What other things do you think could be done to help these patients continue medication treatment?	
**12.** Is there anything else you would like to add that may help me better understand your perspective?	
**13.** Do you have any questions for me?	

### Data analysis

Coding was performed using NVivo software (version 11). To minimize bias, each of the two coders coded the two interviews independently and differences were discussed to determine the initial coding framework. Coding and thematic analysis of remaining interviews was then conducted by one researcher. To increase the validity of the study, themes and subthemes were reviewed and verified by other researchers. Finally, researchers translated themes, subthemes and representative quotations into English. In addition, quotations were edited to correct grammar or remove content of repeated words and stutters.

## Results

We extracted three themes that were likely related to the challenges of medication adherence in patients with severe mental disorders: 1) attitudes towards mental disorders/treatment; 2) inadequate aftercare; and 3) resource shortages. The themes and subthemes were visualized in Table 3.

**Table 3:** Themes and subthemes about challenges of medication adherence in patients with severe mental disorders

Themes	Percentages (%)	Subthemes	Percentages (%)
**1.** Attitudes towards mental disorders/treatment	67.74	**(1)** Inadequate knowledge about mental disorders and treatment	16.13
		**(2)** Insight	19.35
		**(3)** Side effect of treatment	12.90
		**(4)** Stigma	19.36
**2.** Inadequate aftercare environment	45.16	**(1)** Aftercare from family	22.58
		**(2)** Aftercare from community	22.58
**3.** Resource shortage	35.48	**(1)** Financial resource	16.13
		**(2)** Mental health workforce	9.68
		**(3)** Public resource	9.67

### Attitudes towards mental disorders/treatment

#### Inadequate knowledge about mental disorders and treatment

Mental health professionals stated that some patients or their families did not hold a correct or scientific understanding of mental disorders and treatment. As a result, patients or their families thought that medication could be discontinued once the symptoms disappeared after a period of treatment. Additionally, some of these patients might only continue treatments such as Chinese traditional medicine or psychotherapy. Moreover, some patients or families from rural areas believed in supernatural etiologies of mental disorders, which led them to embrace alternative methods that were considered as more effective cures.

*“Some members (patients) thought that they do not need to take medicine, they are normal. ... They think I don’t need to take medicine anymore, I’m alright. They definitely think that not taking medicine is normal and good. Many of them expressed that I don’t need to take medicine tomorrow, I will be fine. I can do whatever I want.” (#19, directors of clubhouse)*

*“Some patients’ families thought that their children were different from other children. They think that previously their children (patients) were stressed, and if they could pay more attention to their children, then they could try to see if it can be effective without taking medicine. (#2, psychiatrist)*

*“Some patients’ families were reluctant to accept that their children (patients) had a mental illness. I saw a patient with severe psychotic symptoms … and suggested his family to take him to hospital. But they thought that their kid was healthy and just possessed by a ghost. They thought he would get better if they used some ritual methods.” (#25, psychological counselor)*

#### Insight

Insight means that the patient’s understanding of their symptoms, illness, prognosis and treatment aligns with that of their healthcare professionals [34]. In general, patients with good insight might be more likely to be compliant with their mental health providers’ plan and maintain medication treatment as prescribed.

Some patients with severe mental disorders, however, had poor insight into their conditions. This lack of awareness probably led many of them to resist medication treatment.

*“We use medication to treat patients. For example, schizophrenia patients with delusions and abnormal behaviors might refuse to accept that they had mental disorder. But, after antipsychotic treatment, they might realize their own mental health problems, be more willing to take antipsychotics, … and finally return to their daily life.” (#1, psychiatrist)*

*“First of all, it is necessary to control their (patients) symptoms, … the drug compliance is better, and the side effects are lighter, so that they might maintain medication treatment more readily.” (#15, researcher of public mental health)*

#### Side effects of treatment

It concerned some patients or their families that medications had side effects that might influence everyday life. For example, some patients might become overweight, or present with an even more severe metabolic syndrome, and other patients might feel drowsy and indolent the whole day which led them to believe that medication was harmful for them. Obviously, they worried about the side effects of medication and neglected to appreciate the drugs’ effectiveness.

*“Some of them (patients) might discontinue medication because it made them fat. … However, most of them relapsed after they discontinue their medication treatment.” (#25, psychological counselor)*

*“Without supervision of parents, some students (patients) might discontinue medication because of side effects, then the symptoms might relapse.” (#30, psychological counselor)*

#### Stigma

Carrying the fear that other people might know about their diagnosis of a mental health disorder, some patients refused to continue taking medication in a public space. For a similar reason, some of the patients or their families would rather receive treatment from internal medicine outpatient departments in general hospitals than outpatient departments of psychiatry in general hospitals or mental hospitals. Therefore, stigma about mental disorders might constrain the use of available mental health resources and might eventually affect the level of medication adherence.

*“There are many reasons that patients with severe mental disorders may be reluctant to take medication, such as stigma, severe side effects or poor insight.” (#15, researcher of public mental health)*

*“We can find certain forms of social discrimination of mental disorders, … some patients’ families thought it might be disgraceful to send patients to a mental health hospital, so they might procrastinate treatment for couple years, ten years, or even a couple decades.” (#10, psychiatrist)*

### Inadequate aftercare

#### Aftercare from families

Almost all patients with severe mental disorders discharged from hospital were taken care of by their families at home. Therefore, the burden of rehabilitation was primarily born by patients’ families. In fact, most of the families needed to supervise the patients to ensure they take medication every day. Sometimes, families even needed to deceive patients into taking medication, although some of the them failed at this.

*“From our point of view, due to traditional notions or conventions, … it is patients’ families that have been faced with the heaviest burden in supporting this kind of (rehabilitation) work. … Therefore, the role and values of patients’ families in managing medication adherence in severe mental disorders cannot be underestimated.” (#6, psychiatrist)*

*“Generally, due to the close relationship among family members in traditional Chinese culture, we will tell patients’ families that patients’ recovery requires a certain period of supervised care and that the medication treatment needs to be continued. And we will also write down some important points, such as when to make a follow-up visit and what kind of medical examination needs to be completed at the next visit, noted on patients’ outpatient medical record book. Lastly, we will tell patients’ families to keep the medication in a secure location.” (#2, psychiatrist)*

#### Aftercare from community

Aftercare from community mostly referred to rehabilitation clubhouses and the 686 Project in this study. In 2004, the 686 Project was initiated to provide comprehensive mental health services to patients with severe mental disorders [35, 36]. This project aimed to improve medication adherence in patients with severe mental disorders by providing free medication routinely and conveniently. Members of clubhouses were patients with severe mental disorders. Referring to patients as members in the clubhouse could reduce stigma and be helpful in creating an appropriate atmosphere for the rehabilitation of patients. The clubhouse staff would ask members everyday if they have taken their medication. And members would discuss with each other the results and practice of taking medication. Additionally, lectures on medication adherence were provided from time to time to create a virtuous cycle for better recovery.

*“In the clubhouse, members would discuss together, … they would often ask other members’ views about medication. … The result of our survey last time showed that 90% of our members continued medication treatment. However, probably 50% of patients or even lower continued medication treatment out in the community. Good medication adherence might be helpful to reduce the relapse rate for patients. … The environment in the clubhouse could help members keep a stable state.” (#17, directors of clubhouse)*

*“For one thing, members will be optimistic (in the clubhouse). For another, members will realize the importance of medication adherence and hold a global perspective about mental disorders as well as medication adherence.” (#18, directors of clubhouse)*

*“They provide them (patients with severe mental disorders) medication for free in community. This is the 686 Project.” (#1, psychiatrist)*

### Resource shortages

#### Financial resources

Some patients might discontinue treatment due to the financial burden. For example, most patients and their families from rural areas or county-level areas would seek out the top hospitals in urban areas in order to pursue better treatment, which might cost them a great expense for transportation and accommodation. Even if the expense of hospitalization could be covered by their healthcare insurance, policies often limited the coverage rate of non-local patients mentioned above. Additionally, expenses incurred in the outpatient department, based on a related policy, were likewise not included in the healthcare insurance plan. Therefore, patients might need to use more expensive medications with more side effects.

*“Although huge amounts of patients come to our outpatient department for treatment, the expense is not entirely covered by healthcare insurance. … Without the subsidy of healthcare insurance, they may take on too great of a financial burden. This is because most of them (patients with severe mental disorders) have no job and they depend on their families to support them financially. Some of them have to travel a great distance to come here, and it costs them many fees for transportation and accommodation, which is even more expensive than the medical expenses.” (#7, psychiatrist)*

*“Some patients (from rural or county-level areas) relapsed many times due to lots of reasons. First of all, previous treatment of this illness already caused them to spend so much money, which made future treatment seem even more unaffordable. … In other words, they were just lacking money (for treatment).” (#10, psychiatrist)*

### Mental health workforce

Several interviewees mentioned that supervision and education of medication adherence required a professional workforce. However, due to limited mental health workforce in the community mental health system and a large flow of patients in outpatient departments in recent years, psychiatrists were often too busy to explain or promote medication adherence to patients in outpatient departments. In addition, it remains difficult to establish a relatively mature system of supervision for mental health management, especially to address medication adherence.

*“There are not enough psychiatrists in our country. During patients’ early stages of severe mental disorders, there needs to be a large related workforce to manage patients when their symptoms are stable and to check if they take medication regularly. All of this labor requires a relatively large mental health workforce to support it. … If there are social workers, and an adequate mental health workforce in this field, they can supervise the symptoms and the treatment of patients. … However, there is a shortage in our country.” (#5, psychiatrist)*

#### Social resources

Social resources, especially from government sources, are likely to improve psychiatric patients’ medication adherence. Currently, the management of patients with mental disorders, especially those in the community, is underdeveloped. However, the government did implement some measures, such as the 686 Project, a series of mental health policies and various mental health services designed to meet the needs of psychiatric patients in the community.

*“In our country, most mental health professionals only focus on the management of patients in mental health hospitals. This may require the government to implement measures allowing psychiatrists to go to communities and to treat patients there. And it has been promoting such measures. For example, psychiatrists, nurses and other related mental health professionals may organize a team, then go to communities and distribute medications to patients with severe mental disorders. This is the 686 Project which is in process.” (#1, psychiatrist)*

*“The most important thing is that they (patients with severe mental disorders) need social support. … It is a tough issue that has caused some of them to relapse many times. Therefore, in order to see more positive results in medication adherence, the government needs to actively intervene and our society needs to adopt some measures.” (#10, psychiatrist)*

## Discussion

In our study, we identified that attitudes towards mental disorders/treatment, inadequate aftercare environment, and resource shortages might affect medication adherence in patients with severe mental disorders in the Chinese context.

### Attitudes towards mental disorders/treatment

Multiple studies have shown that psychiatric patients’ or their family members’ attitudes towards mental disorders and treatment can impact medication adherence to various degrees [16, 26, 37, 38]. Additionally, results of a cross-sectional study revealed that Asians held more negative beliefs towards taking medication compared to Europeans [39]. In our qualitative research, we found that knowledge about mental disorders and treatment, patients’ levels of insight, awareness of the side effects of the treatment, and social stigma could also influence psychiatric patients’ medication adherence.

In the Chinese context, patients or their family members’ inadequate knowledge about mental disorders may lead to misconceptions about the care received, which results in poor medication taking behaviors. Consistent with previous studies, we found inadequate knowledge about mental disorders and treatment being one of the risk factors of medication nonadherence [17, 18]. In China, a large number of patients with mental health disorders and their family members perceive that continuous psychotropic medication use can be harmful and addictive [16, 40]. Many of them sought help from traditional Chinese medicine or superstitious methods [29, 41]. Additionally, previous literature suggested that poor insight into mental disorders, concerns over the side effects of medications, and perceived stigma about mental disorders constrained proper adherence to medications [22, 25, 42]. First, patients with severe mental disorders who have poor insight into their conditions are less willing to continue medication or to follow their psychiatrist’s medication plan rigorously [25]. Second, psychotropic medications like antipsychotics or antidepressants may introduce certain side effects, such as drowsiness, weight gain, diabetes, or sexual dysfunction, which might impact patients’ daily functioning and directly decrease their medication adherence [43]. Last, as stigma toward patients with severe mental disorders is quite common in China [44, 45], Saxena et al. investigated that stigma towards mental disorders could constrain the use of available mental health resources [46]. Worried about being discriminated by others, many patients might avoid taking medications in public space or even discontinue their medications. Even their family members might also discourage them for the same reason [45, 47].

Several researchers concluded that collaborative decision making, also termed therapeutic alliance, enables patients with mental disorders to be more involved in choosing their medication regime, which is a key factor for better medication adherence [42, 48, 49]. A shared decision-making process could empower patients to actively communicate with their mental health providers and help providers be aware of the difficulties that a patient faces during treatment. As a result of this therapeutic collaboration, patients and their family members’ attitudes towards mental health and treatment would be more positive and their stigma could be reduced as well. Moreover, with better communication with their psychiatrists, patients might be more satisfied with their treatment and report fewer side effects due to timely management [22]. Noordraven et al. demonstrated that insight was less strongly associated with medication adherence than motivation for treatment, so it may be more important to improve motivation as the first step in patients with poor insight as the recovery of insight was difficult and time consuming [24]. In this case, psychoeducation could also be employed to spread the knowledge about mental disorders and treatment, enhance insight, and manage side effects, and reduce stigma, which may ultimately improve medication adherence [14, 26, 50–53].

### Inadequate aftercare

Aftercare refers to mental health care given outside the hospital. It consists of supervision of medication use, follow-up of recurrence, rehabilitation of social function as well as work skills training. Due to the clinical characteristics of severe mental disorders (e.g., chronic course and impaired social function), aftercare is one of the key factors that determines the prognosis of severe mental disorders. In China, patients with mental disorders mostly live with or have close connection with their family members, so the burden of aftercare often falls on the latter. However, as their family members’ knowledge of and time dedicated to aftercare is often limited, the aftercare burden experienced by patients’ family members was inversely correlated with patients’ medication adherence, as shown in a previous study [54]. Another research study in China also demonstrated that patients with severe mental disorders heavily relied on the aftercare from their family members [55], which urged the spread and development of community-based mental health programs for patients and caregivers. As a large part of community-based mental health services still requires family participation [56], it is understood that aftercare from patients’ families and rehabilitation both play significant roles in maintaining medication adherence [14].

In China, the government has launched a program in 2004 called the 686 Project to improve community-based mental health services. The 686 Project was initiated to provide comprehensive monitoring, key mental health treatment, rehabilitation, and prevention services to patients with severe mental disorders from a wide range of urban and rural communities [35, 36, 57, 58]. Additionally, an increasing number of rehabilitation services have been emerging including the rehabilitation clubhouse model, farming programs, and workstation programs [50, 59, 60]. In this qualitative research, we mainly focused on the rehabilitation clubhouse as one of the community-based mental health services currently offered. Previous literature identified the positive influence of mental health rehabilitation services on prevention, treatment, and rehabilitation of mental disorders [50, 61]. Consistent with these studies, we found that mental health aftercare played an essential role in medication adherence in patients with severe mental disorders. However, the aftercare offered by patients’ family members and their communities is currently inadequate. Therefore, besides improving caregivers’ knowledge of mental health aftercare, multiple types of community-based mental health services or innovative service models are needed. For example, Xu et al. demonstrated that mobile text messaging was an effective way to integrate family-based aftercare into community-based aftercare, leading to improvements in medication adherence in patients with severe mental disorders [62].

### Resource shortages

In the present study, we found that the shortage of financial resources, the mental health workforce, and social resources may be among the main factors that affected medication adherence in patients with severe mental disorders. In China, there are currently many challenging issues causing this resource shortages for mental health. For example, in considering financial resource shortages, the health insurance system in China has developed separate reimbursement models in urban and rural areas, which might increase inequity of mental health resources offered across different areas [63]. Even with health insurance coverage, many families cannot afford the expenses of treatment for severe mental disorders due to the chronicity of the disease. Therefore, some of them used less expensive medications with more side effects, which might increase the risk of medication nonadherence. Second, medication adherence in patients with severe mental disorders requires adequate supervision and education from mental health professionals. However, in 2015, there were 27,733 licensed psychiatrists (2.02 per 100,000 population) and 57,591 psychiatric nurses (4.19 per 100,000 population) in China [36]. In contrast, according to the mental health atlas of WHO in 2014 [64], there were 7 psychiatrists per 100,000 population and 24.1 psychiatric nurses per 100,000 population in European regions, while there were 0.1 psychiatrists per 100,000 population and 0.6 psychiatric nurses per 100,000 population in African regions. This stretched workforce may affect the medication adherence in patients with severe mental disorders and may limit the access for patients to seek help. The limited mental health workforce constitutes an important barrier to proper mental health service delivery, which in turn, slows down the process of services integration [65]. Finally, different levels of social resources were scarce or fragmented in distribution, including mental health policies, non-governmental organization support, mental health services (especially community-based services) and support from social networks. All of these may provide substantial support in the integration of mental health resources, spread of psychoeducation, case management and so on, which are vital to improve the medication adherence in patients with severe mental disorders. In a word, mental health resource scarcity may constrain the availability of mental health services and essential medication for patients, which eventually hinders the medication adherence in patients with severe mental disorders.

In the past two decades, the Chinese government implemented several effective interventions to strengthen and integrate mental health resources. First, the 686 Project, initiated in 2004, was a comprehensive service delivery model aiming to integrate hospital services, community case management, prevention and monitoring services, treatment, and rehabilitation services [36]. Second, as suggested by one of our previous studies, the rehabilitation clubhouse model has been in use in Hunan Province, China, and both the central and provincial governments took active steps to promote its development in more areas [50, 66]. Third, alongside strategies to improve undergraduate and graduate medical education [67], one of the most efficient steps taken to advance mental health workforce development was the psychiatrist licensing program announced by the National Family Planning and Health Commission in 2015 [68]. This program allows physicians who were already in practice to receive further training in psychiatry and to be licensed as psychiatrists. Finally, a series of mental health policies has been launched since 2000 [69], including the Mental Health Law of the People’s Republic of China was released on 2011 and came into effect from 2013 [19, 70, 71]. Although the government has initiated a series of steps to reinforce mental health resources, their uneven distribution still persists [72]. Furthermore, Thornicroft et al. demonstrated that a balanced approach in planning community-based and hospital-based mental health services was effective in both urban and rural areas [73]. By gradually strengthening community-based services, medication adherence is expected to improve in patients with severe mental disorders that often require more supervision and nearby assistance.

Adequate mental health resources are one of the foundations to improving medication adherence in patients with severe mental disorders. Due to the challenges of lacking mental health resources that China has confronted, it is vital to implement measures that are well adapted to the local context. First, the government should mobilize existing mental health resources, both to improve access to and increase the capacity of mental health services. Second, in order to reduce inequity and fragmentation of mental health resources between urban and rural areas, we can designate mental health professionals from urban areas to rural areas periodically as well as make use of telemedicine. For example, online consultation is considered efficient in balancing the distribution of mental health resources between urban and rural areas, and even between first-tier cities and second-tier cities. Third, to implement mental health policies efficiently from top to bottom, local-level execution and national-level promotion are equally important. Fourth, despite the heavy economic burden of mental disorders, equitable allocation of financial resources in mental health should still be emphasized, as it not only improves medication adherence in patients with severe mental disorders but also reduces the overall cost of mental health. Finally, non-psychiatric mental health professionals, including psychologists, counselors, social workers, and occupational therapists are also currently in high demand [68]. Diversifying the mental health workforce is therefore urgently needed to relieve psychiatrists’ and psychiatric nurses’ burden and provide comprehensive care.

### Implications

The issue of medication nonadherence in patients with severe mental disorders has its own unique circumstances in different settings, and this qualitative study provided evidence based on the Chinese context to illuminate the effects of local conditions on clinical work. In clinical work, psychiatrists and nurses should pay more attention to the therapeutic alliance with patients and patients’ families, which can assist them in identifying patients’ insight and addressing the side effects of treatment in a timely manner to improve clinical outcomes. Moreover, psychiatrists and nurses should strengthen psychoeducation regarding mental disorders and treatment in patients and their families to reduce their misconceptions. Considering the importance of psychiatric community rehabilitation in medication adherence, governments should establish local community-based mental health services. As some mental health projects (e.g., 686 Project) and mental health policies have achieved certain improvement in medication adherence in some areas of China, governments should promote the successful and culturally adapted mental health models to other areas in China as well as integrate the mental health workforce at all levels to implement the mental health models efficiently. With the aforementioned effective steps, the future mental health system may improve the medication adherence in patients with severe mental disorders and reduce the economic burden for patients and society at large in China.

### Limitation

One limitation of this qualitative study is that the sample size is relatively small. However, saturation did occur during the process of participant enrollment, which suggested the adequacy of the sample size in this particular study. In addition, there is potential bias caused by the snowballing sampling method which is a purposive sampling. Nevertheless, as purposive sampling is widely used in qualitative studies to identify and select information-rich participants related to the phenomenon of interest, snowballing sampling method is a highly-efficient way to identify potential participants from whom researchers may explore more useful information [31]. Another limitation is that the present study only looked at rehabilitation clubhouses and the 686 Project as examples of community-based mental health services, which may not reflect the whole picture nationally. The occurrence of data saturation in our case may be due to the location of our research, since the rehabilitation clubhouses and 686 Project were both in Hunan Province and more successful in promoting medication adherence than other forms of mental health aftercare in community settings. Along the same lines, in considering the provincial differences in mental health services across the country, the fact that our findings were entirely generated from Hunan province may limit the data applicability when generalized to the national level. But given that we have investigated medication adherence in patients with severe mental disorders in both urban areas and rural settings, it may still provide useful information to researchers in the field. Furthermore, it may also provide support for this kind of research in other Asian countries with a cultural context similar to that of China. However, as a qualitative study, there may be limited generalizability beyond our setting.

## Conclusion

In summary, the present study focused on the perspectives of mental health professionals to identify factors influencing medication adherence among patients with severe mental health disorders in Hunan Province, China. Our findings provide practical advice for mental health providers on the promotion of medication adherence among patients with severe mental disorders. Moreover, this locally driven research also suggests culturally tailored models that improve the management of patients with severe mental disorders in order to reduce economic burden on individual and societal level. Future researchers could further explore the medication adherence among patients with severe mental disorders in other cultural contexts.

## Data Availability

The data generated and analyzed during the current study are not publicly available to protect the anonymity of the participants. Materials may be available from the corresponding author upon reasonable request.

## Abbreviations

WHO: World Health Organization
DALYs: Disability-adjusted life-year
